# Prevalence of common and rare ophthalmic findings in adults attending a medical survey institute

**DOI:** 10.1007/s10792-024-03026-8

**Published:** 2024-02-09

**Authors:** Daphna Landau Prat, Noa Kapelushnik, Ofira Zloto, Ari Leshno, Eyal Klang, Sigal Sina, Shlomo Segev, Mattan Arazi, Shahar Soudry, Guy J. Ben Simon

**Affiliations:** 1 Goldschleger Eye Institute, Sheba Medical Center, Tel Hashomer, Israel; 2 Talpiot Medical Leadership Program, Sheba Medical Center, Tel Hashomer, Israel; 3 Faculty of Medicine, Tel-Aviv University, Ramat Aviv, Israel; 4 The Sami Sagol AI Hub, ARC Innovation Center, Sheba Medical Center, Tel Hashomer, Israel; 5 Institute for Medical Screening, Sheba Medical Center, Tel Hashomer, Israel; 6 Timna, Ministry of Health, Jerusalem, Israel

**Keywords:** Ophthalmic data, Big data, Cataract prevalence, Optic pit

## Abstract

**Purpose:**

To examine the ophthalmic data from a large database of people attending a general medical survey institute, and to investigate ophthalmic findings of the eye and its adnexa, including differences in age and sex.

**Methods:**

Retrospective analysis including medical data of all consecutive individuals whose ophthalmic data and the prevalences of ocular pathologies were extracted from a very large database of subjects examined at a single general medical survey institute.

**Results:**

Data were derived from 184,589 visits of 3676 patients (mean age 52 years, 68% males). The prevalence of the following eye pathologies were extracted. Eyelids: blepharitis (n = 4885, 13.3%), dermatochalasis (n = 4666, 12.7%), ptosis (n = 677, 1.8%), ectropion (n = 73, 0.2%), and xanthelasma (n = 160, 0.4%). Anterior segment: pinguecula (n = 3368, 9.2%), pterygium (n = 852, 2.3%), and cataract or pseudophakia (n = 9381, 27.1%). Cataract type (percentage of all phakic patients): nuclear sclerosis (n = 8908, 24.2%), posterior subcapsular (n = 846, 2.3%), and capsular anterior (n = 781, 2.1%). Pseudophakia was recorded for 697 patients (4.6%), and posterior subcapsular opacification for 229 (0.6%) patients. Optic nerve head (ONH): peripapillary atrophy (n = 4947, 13.5%), tilted disc (n = 3344, 9.1%), temporal slope (n = 410, 1.1%), ONH notch (n = 61, 0.2%), myelinated nerve fiber layer (n = 94, 0.3%), ONH drusen (n = 37, 0.1%), optic pit (n = 3, 0.0%), and ON coloboma (n = 4, 0.0%). Most pathologies were more common in males except for ONH, and most pathologies demonstrated a higher prevalence with increasing age.

**Conclusions:**

Normal ophthalmic data and the prevalences of ocular pathologies were extracted from a very large database of subjects seen at a single medical survey institute.

## Introduction

Normal data are the cornerstone of medical science by providing reference points for physical examinations and enabling the identification of disease and pathological states. The prevalence of ophthalmic findings varies widely, depending upon various factors, such as age, sex, race, lifestyle, and underlying medical conditions [1]. Understanding the prevalence of ocular findings is critical for clinicians to make appropriate diagnoses, develop treatment plans, and prevent vision loss. Previous studies have provided ophthalmic measures in relatively large population-based cohorts of patients, some of the well-known among them include the Beijing Eye Study [2], the Beaver Dam Eye Study [3], The Blue Mountains Eye Study [4], the Age-Related Eye Disease Study 2 [5], and others [6]. The sample size in those studies ranged between 3654 [4] and 81,148 [6] individuals.

This paper aims to provide an overview of the prevalence of ophthalmic findings in people who voluntarily attended a health screening. Participants had a comprehensive eye examination including recording a full range of ophthalmic diagnoses. Our objective was to create a single report that includes an analysis of various ophthalmic variables and observations, encompassing both the prevalence of common findings and the exploration of rare occurrences.

## Methods

We analyzed the medical records of individuals examined at the Institute of Medical Survey at the Sheba Medical Center during the 20 years between May 2001 and August 2020. The data included 195,969 visits of 37,692 adults who had been examined by an ophthalmologist. The retrieved data on the comprehensive ophthalmic examination findings included those of the anterior and posterior segments. The prevalence of glaucoma mentioned in the results section was extracted from the database using artificial intelligence analysis, as detailed in a previous study [7].

### Study population

All patients were healthy individuals who ambulatorily attended the Medical Survey Institute in which individuals electively undergo a series of systemic and ocular screening examinations and are referred for additional tests as needed. These general examinations were performed by various subspecialties, such as dermatology, ophthalmology, internal medicine, cardiology, and others. Demographic data, vital signs, and general blood tests are routinely obtained. This institution is independent of the main hospital services, such as medical or surgical clinics, hospital wards, and the emergency room. Many attendees willingly undergo yearly screening tests, and the costs of these elective tests are commonly covered by their insurance or employers. Thus, the current database includes no patients with acute medical issues or any other emergencies.

The database included visits of 36,762 adults who had undergone comprehensive ophthalmic and systemic evaluations in the Institute. The retrieved information included demographic data (age, sex, family status, country of birth, and nationality), vital signs, detailed ergometry examination results, blood test results (complete blood count [CBC], chemistry, and additional ones as needed), and final diagnoses (hypertension, diabetes mellitus, etc.). The ophthalmic examination included a detailed anterior and posterior segment evaluation. The prevalence of each ophthalmic finding was calculated and additionally stratified by sex and age.

### Data preparation

Each input file was integrated into the research environment and fully validated against the source before the data analysis. The ocular examination data were derived from the recorded field. Various variables originally included free text that contained information in a non-uniform manner. Text analysis was performed on a graded process that uses regular expression (regex) techniques. For example, the “eyelid” field for each patient may include a single word, such as “normal”, or it can include multiple texts, such as “blepharitis, dermatochalasia and ptosis with mild brow ptosis”. This process resulted in the creation of a broad semantic layer of dozens of ophthalmological metrics, including a wide range of ocular findings.

### Statistical analysis

Data analysis of the study variables was expressed in descriptive statistics of characteristic percentiles, including ranges, medians, and interquartile ranges (IQR) to represent the distributions of the continuous variables, and in frequencies and percentages for indicative or categorical variables. Kruskal–Wallis tests were performed for continuous features, and Chi-squared tests were performed for categorical features. Outliers were identified using descriptive analyses and boxplot charts for the continuous variables. Only the right eye was included in the statistical analysis in order to avoid the influence of inter-eye correlations. Descriptive data and analyses were based upon the findings during the final visit.

### Research ethics approval

The described research adhered to the tenets of the Declaration of Helsinki, the study was HIPAA-compliant, and Institutional Review Board (IRB) approval was obtained. Patient consent was waived for this anonymized retrospective study.

## Results

The data were derived from 184,589 visits of 36,762 individuals (68% males and 32% females) with a mean age of 52 years (59% were 40 to 60 years old). The prevalence of the following pathologies derived from the general ophthalmic data analysis were extracted. Eyelids: blepharitis (n = 4885, 13.3%), dermatochalasis (n = 4666, 12.7%), upper eyelid ptosis (n = 677, 1.8%), lower eyelid ectropion (n = 73, 0.2%), and xanthelasma (n = 160, 0.4%). Anterior segment: pinguecula (n = 3368, 9.2%), pterygium (n = 852, 2.3%), and cataract or pseudophakia (n = 9381, 27.1%). Cataract type (percentage of all phakic patients): nuclear sclerosis (n = 8908, 24.2%), posterior subcapsular (n = 846, 2.3%), and capsular anterior (n = 781, 2.1%). Pseudophakia was present in 1697 patients (4.6%), and 229 (0.6%) patients had posterior subcapsular opacification. Optic nerve head (ONH): peripapillary atrophy (PPA; n = 4947, 13.5%), tilted optic disc (n = 3344, 9.1%), temporal slope (n = 410, 1.1%), ONH notch (n = 61, 0.2%), myelinated nerve fiber layer (MNFL; n = 94, 0.3%), ONH drusen (ONHD; n = 37, 0.1%), optic pit (n = 3, 0.0%), and ON coloboma (n = 4, 0.0%). Retina and vitreous: macular pigmentary changes (n = 4496, 12.2%), macular drusen (n = 1159, 3.2%), and 1.6.%, arteriovenous (A-V) crossing (n = 1189, 3.2%). Glaucoma prevalence was assessed in a subgroup of 27,517 patients; 633 patients (2.3%) were diagnosed as having glaucoma. In this subgroup, the mean VA was 20/21, IOP 14.4 ± 2.84 mm Hg, and CDR 0.28 ± 0.16.

### Sex differences in pathology prevalence

Significant sex differences were found in the following parameters (univariate analysis, chi-square): blepharitis (males 16% vs. females 6%, *P* < 0.001), dermatochalasis (15% vs. 9%, *P* < 0.001), upper eyelid ptosis (2.1% vs. 1.4%, *P* < 0.001), ectropion (0.3% vs. 0.07%, *P* < 0.001), pterygium (2.7% vs. 1.5%, *P* < 0.001), and pinguecula (10% vs. 7%, *P* < 0.001). The sex differences in lens status were: posterior chamber IOL (males 5% vs females 4%, *P* < 0.001), cataract all types (29% vs. 23%, *P* < 0.001), nuclear sclerosis (27% vs. 22%, *P* < 0.001), posterior subcapsular (2.7% vs. 2%, *P* < 0.001), and capsular anterior (2.4% vs. 2%, *P* = 0.02). Sex differences in optic disc pathologies included: tilted (9% vs. 9.7%, *P* = 0.02), and normal cup (cup-to-disc ratio < 0.5) (88.1% vs. 90.7%, *P* < 0.001). In addition, males had a higher incidence of ON temporal slope (1.24% vs. 0.89% for females, *P* = 0.004, chi-square). No significant sex differences were observed for PPA, MNFL notch, MNFL, optic pit, ON coloboma, or ONHD. Finally, sex differences were noted in macular pigmentary changes (13.7% males vs. 9.6% females, *P* < 0.001), drusen (3.4% vs. 2.7%, *P* = 0.001), epiretinal membrane (ERM) (1.7% vs. 1.3%, *P* = 0.003), and A-V crossings (4.0% vs. 1.7%, *P* < 0.001).

### Age differences

**Fig. 1:**
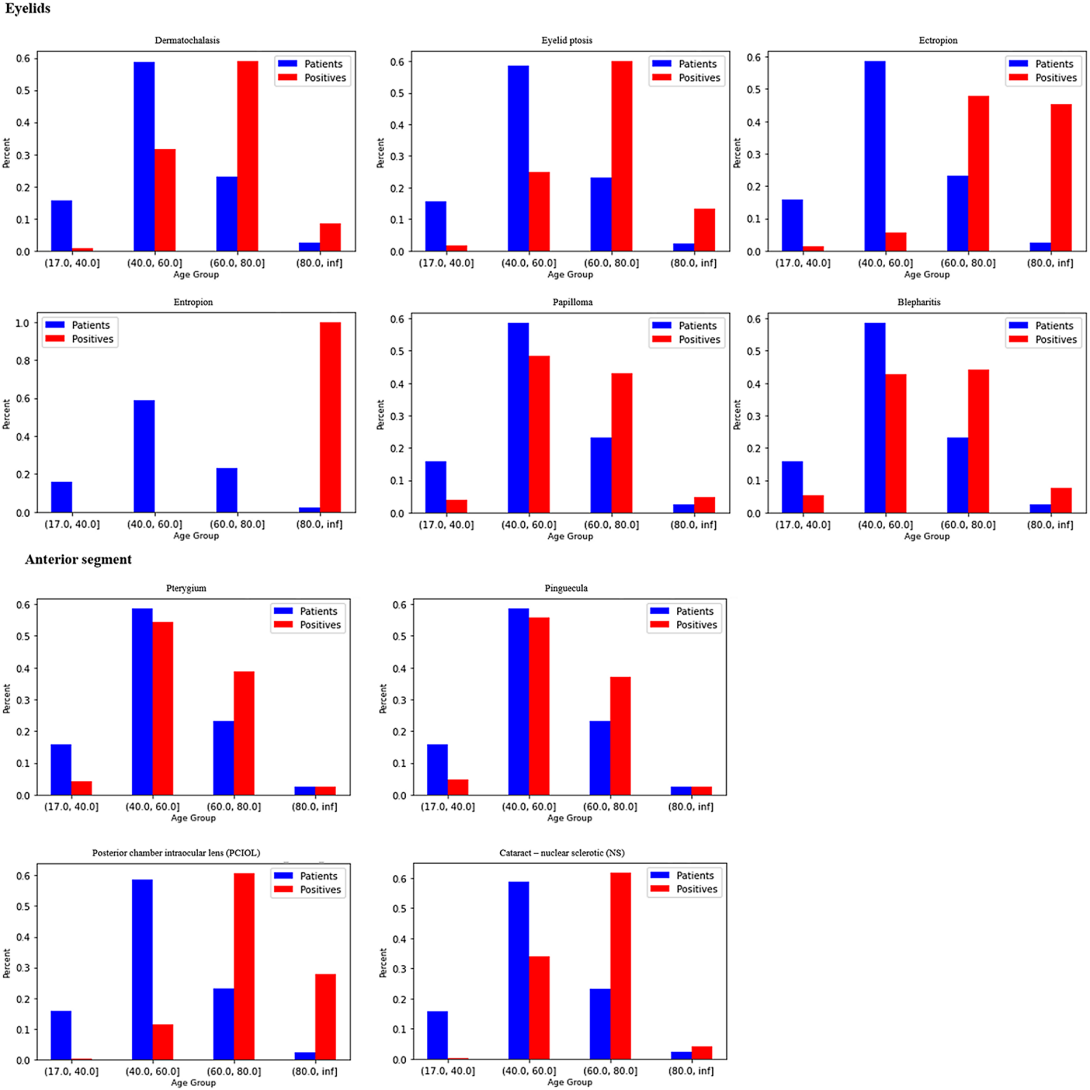
Eyelid and anterior segment findings at different age quartiles among 36,762individuals. Blue bars represent their age distribution in different age quartiles and are identical in all charts. Red bars represent the age group distribution of those who were positive for each pathology

**Fig. 2:**
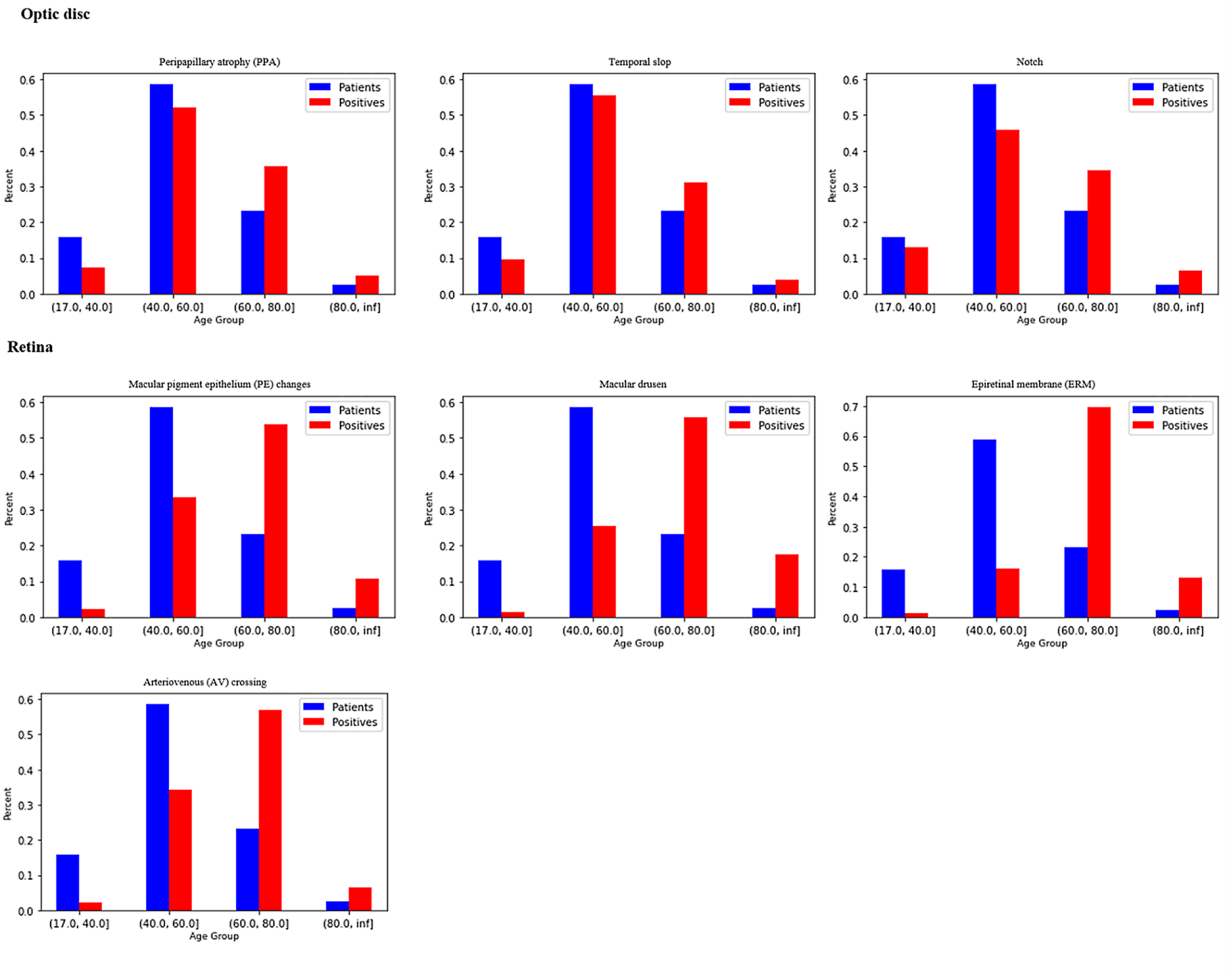
Optic nerve and retinal findings at different age quartiles among 36,762 individuals. Blue bars represent their age distribution in different age quartiles and are identical in all charts. Red bars represent the age group distribution of those who were positive for each pathology

Data were analyzed according to the age quartiles 18–40, 40–60, 60–80, and over 80 years. Variables that increased in incidence with age included eyelid malposition (dermatochalasis, ptosis, ectropion, entropion; papilloma, and blepharitis), while anterior segment pathologies (pinguecula, pterygium, and cataracts of all types) showed a similar trend. Posterior segment pathologies (CD ratio, PPA, ON temporal slope, ON notch, macular pigmentary changes, drusen, ERM, AMD, and A-V crossing) demonstrated an increased prevalence with aging. Table 1, Fig. 1, and Fig. 2 describe the differences in ophthalmic findings according to age groups.

**Table 1:** Prevalence of ophthalmic findings by age group in 36,762 adults examined at the Sheba Medical Center between 2001 and 2020

Variable	Value	Missing (n)	Overall	[18.0, 40.0y]	(40.0, 60.0y]	(60.0, 80.0]	(80.0, inf]	*P*-Value	Test
N			36,762	5,781	21,578	8514	889		
*Eyelids*									
Ectropion, n (%)	0	96	36,593 (99.801)	5772 (99.983)	21,537 (99.981)	8438 (99.587)	846 (96.246)	< 0.001	Chi-squared (warning: expected count < 5)
	1		73 (0.199)	Less_15	Less_15	35 (0.413)	33 (3.754)		
Papilloma, n (%)	0	96	35,575 (97.024)	5730 (99.255)	21,013 (97.549)	8004 (94.465)	828 (94.198)	< 0.001	Chi-squared
	1		1091 (2.976)	43 (0.745)	528 (2.451)	469 (5.535)	51 (5.802)		
Xanthelasma, n (%)	0	96	36,506 (99.564)	5768 (99.913)	21,441 (99.536)	8419 (99.363)	878 (99.886)	< 0.001	Chi-squared (warning: expected count < 5)
	1		160 (0.436)	Less_15	100 (0.464)	54 (0.637)	Less_15		
Blepharitis, n (%)	0	96	31,781 (86.677)	5513 (95.496)	19,453 (90.307)	6313 (74.507)	502 (57.110)	< 0.001	Chi-squared
	1		4885 (13.323)	260 (4.504)	2088 (9.693)	2160 (25.493)	377 (42.890)		
Entropion, n (%)	0	96	36,665 (99.997)	5773 (100.000)	21,541 (100.000)	8473 (100.000)	878 (99.886)	< 0.001	Chi-squared (warning: expected count < 5)
	1		Less_15	Less_15	Less_15	Less_15	Less_15		
Dermatochalasis, n (%)	0	96	32,000 (87.274)	5742 (99.463)	20,062 (93.134)	5715 (67.450)	481 (54.721)	< 0.001	Chi-squared
	1		4666 (12.726)	31 (0.537)	1479 (6.866)	2758 (32.550)	398 (45.279)		
Ptosis, n (%)	0	96	35,989 (98.154)	5762 (99.809)	21,372 (99.215)	8066 (95.197)	789 (89.761)	< 0.001	Chi-squared
	1		677 (1.846)	11 (0.191)	169 (0.785)	407 (4.803)	90 (10.239)		
*Anterior chamber*									
Pterygium, n (%)	0	112	35,798 (97.675)	5737 (99.376)	21,064 (97.849)	8139 (96.092)	858 (97.500)	< 0.001	Chi-squared
	1		852 (2.325)	36 (0.624)	463 (2.151)	331 (3.908)	22 (2.500)		
Pinguecula, n (%)	0	112	33,282 (90.810)	5613 (97.228)	19,652 (91.290)	7224 (85.289)	793 (90.114)	< 0.001	Chi-squared
	1		3368 (9.190)	160 (2.772)	1875 (8.710)	1246 (14.711)	87 (9.886)		
*Lens*									
Pseudophakic, n (%)	0	452	34,613 (95.326)	5740 (99.965)	21,139 (99.095)	7347 (87.694)	387 (45.105)	< 0.001	Chi-squared
	1		1697 (4.674)	Less_15	193 (0.905)	1031 (12.306)	471 (54.895)		
Lens PCO, n (%)	0	452	36,081 (99.369)	5742 (100.000)	21,302 (99.859)	8251 (98.484)	786 (91.608)	< 0.001	Chi-squared
	1		229 (0.631)	Less_15	30 (0.141)	127 (1.516)	72 (8.392)		
Lens clear, n (%)*	0	2,149	9381 (27.103)	52 (0.906)	3305 (15.635)	5661 (77.052)	363 (93.798)	< 0.001	Chi-squared
	1		25,232 (72.897)	5688 (99.094)	17,834 (84.365)	1686 (22.948)	24 (6.202)		
*Cataract type*									
Nuclear sclerotic (NS), n (%)*	0	2,149	25,705 (74.264)	5717 (99.599)	18,122 (85.728)	1838 (25.017)	28 (7.235)	< 0.001	Chi-squared
	1		8908 (25.736)	23 (0.401)	3017 (14.272)	5509 (74.983)	359 (92.765)		
NS type, n (%)†	1	27,854	7827 (87.865)	23 (100.000)	2955 (97.945)	4709 (85.478)	140 (38.997)	< 0.001	Chi-squared (warning: expected count < 5)
	2		868 (9.744)	Less_15	53 (1.757)	675 (12.253)	140 (38.997)		
	3		199 (2.234)	Less_15	Less_15	121 (2.196)	71 (19.777)		
	4		Less_15	Less_15	Less_15	Less_15	Less_15		
Posterior subcapsular cataract (PSC), n (%)*	0	2,149	33,767 (97.556)	5714 (99.547)	20,831 (98.543)	6903 (93.957)	319 (82.429)	< 0.001	Chi-squared
	1		846 (2.444)	26 (0.453)	308 (1.457)	444 (6.043)	68 (17.571)		
Cortical anterior cataract (CA), n (%)*	0	2,149	33,832 (97.744)	5731 (99.843)	20,979 (99.243)	6813 (92.732)	309 (79.845)	< 0.001	Chi-squared
	1		781 (2.256)	9 (0.157)	160 (0.757)	534 (7.268)	78 (20.155)		
*Optic nerve*									
Tilted disc, n (%)	0	508	32,910 (90.776)	5295 (92.263)	19,402 (90.974)	7453 (89.375)	760 (89.517)	< 0.001	Chi-squared
	1		3344 (9.224)	444 (7.737)	1925 (9.026)	886 (10.625)	89 (10.483)		
Peripapillary atrophy (PPA), n (%)	0	508	31,307 (86.355)	5373 (93.623)	18,757 (87.950)	6575 (78.846)	602 (70.907)	< 0.001	Chi-squared
	1		4947 (13.645)	366 (6.377)	2570 (12.050)	1764 (21.154)	247 (29.093)		
Temporal slop, n (%)	0	508	35,844 (98.869)	5700 (99.320)	21,100 (98.936)	8211 (98.465)	833 (98.115)	< 0.001	Chi-squared
	1		410 (1.131)	39 (0.680)	227 (1.064)	128 (1.535)	16 (1.885)		
Notch, n (%)	0	508	36,193 (99.832)	5731 (99.861)	21,299 (99.869)	8318 (99.748)	845 (99.529)	0.018	Chi-squared (warning: expected count < 5)
	1		61 (0.168)	Less_15	28 (0.131)	21 (0.252)	Less_15		
Myelinated retinal nerve fiber layer, n (%)	0	508	36,160 (99.741)	5721 (99.686)	21,273 (99.747)	8320 (99.772)	846 (99.647)	0.73	Chi-squared (warning: expected count < 5)
	1		94 (0.259)	18 (0.314)	54 (0.253)	19 (0.228)	Less_15		
Optic pit, n (%)	0	508	36,251 (99.992)	5738 (99.983)	21,325 (99.991)	8339 (100.000)	849 (100.000)	0.712	Chi-squared (warning: expected count < 5)
	1		Less_15	Less_15	Less_15	Less_15	Less_15		
Optic disc coloboma, n (%)	0	508	36,250 (99.989)	5739 (100.000)	21,323 (99.981)	8339 (100.000)	849 (100.000)	0.424	Chi-squared (warning: expected count < 5)
	1		Less_15	Less_15	Less_15	Less_15	Less_15		
Optic nerve head drusen, n (%)	0	508	36,217 (99.898)	5730 (99.843)	21,309 (99.916)	8330 (99.892)	848 (99.882)	0.496	Chi-squared (warning: expected count < 5)
	1		37 (0.102)	Less_15	18 (0.084)	Less_15	Less_15		
*Retina*									
Macular retinal pigment epithelium changes, n (%)	0	603	31,663 (87.566)	5618 (98.165)	19,778 (92.959)	5902 (70.972)	365 (43.246)	< 0.001	Chi-squared
	1		4496 (12.434)	105 (1.835)	1498 (7.041)	2414 (29.028)	479 (56.754)		
Macular drusen, n (%)	0	603	35,000 (96.795)	5706 (99.703)	20,982 (98.618)	7670 (92.232)	642 (76.066)	< 0.001	Chi-squared
	1		1159 (3.205)	17 (0.297)	294 (1.382)	646 (7.768)	202 (23.934)		
Arteriovenous crossings, n (%)	0	641	34,932 (96.708)	5690 (99.528)	20,840 (98.094)	7636 (91.845)	766 (90.651)	< 0.001	Chi-squared
	1		1189 (3.292)	27 (0.472)	405 (1.906)	678 (8.155)	79 (9.349)		

^*^% of those who did not undergo cataract extraction

^†^NS type—nuclear sclerotic cataract grading from 1 to 4

## Discussion

Our study describes selected ophthalmic findings derived from a very large-scale database of a people attending the medical survey institute. The participants of the current study were generally healthy individuals who voluntarily underwent systemic and ocular screening tests. They all underwent a comprehensive ophthalmic evaluation that included ocular adnexal, anterior segment and dilated fundus examinations, thus providing us with a unique opportunity to describe both common and rare ophthalmic conditions. In the following sections, we will discuss the primary findings and compare them to the existing body of knowledge.

### Adnexa

Dermatochalasis and blepharitis were the most common eyelid conditions, with a prevalence of 13% each, ranging from 0.5% to 4.5% in the 18– to 40-year-old group and up to 45% in the older groups. Although dermatochalasis is very common among the elderly, there are very few reports on its actual prevalence. In contrast, the prevalence of blepharitis is well-described, and it can reportedly be as common as 48% to 58% in patients 60 years of age or older [8]. Our population was less affected, most probably because our patient population tended to be younger. Of note, previous reports observed that the prevalence of Demodex blepharitis was higher among patients treated with anti-inflammatory medications for dry eye [8]; however, the disease is underdiagnosed and should be specifically screened in any ophthalmic assessment.

### Blepharoptosis

The prevalence of ptosis in our cohort of patients was 1.8% overall and 10% in patients older than 80 years of age. Various population-based studies described similar numbers ranging from 4.7% to 13.5%, [1, 9–11] demonstrating the widespread nature of ptosis among the elderly. Ptosis results in the presence of reduced visual field (VF) and tired appearance, as well as reduced independence, depression and anxiety, and reduced health-related QOL [12]. VF loss is also associated with recurrent falls and a higher risk of hip and non-hip fractures [13]. Ptosis correction surgery is associated with a higher functional index and significant improvements in activities of daily living [14].

### Cataract

Cataracts are a significant public health concern, being as they are the leading cause of blindness worldwide [15]. Several risk factors are associated with cataract development, including age, genetics, exposure to ultraviolet radiation, smoking, diabetes, and certain medications [16]. In the current study, the anterior segment pathologies of cataract and pseudophakia were by far the most common, with an overall prevalence of 30.1%, from 1% in younger patients and rising to 94% in patients older than 80 years. These findings are similar to those of previous studies that described an average cataract prevalence of 17.2%, with most patients being older than 60 years [17]. We found a higher prevalence of both cataracts and pseudophakia among males, although other studies have shown that it may be more common in women [18]. One recent study investigated the current prevalence of pseudophakia in a well-defined United States population, and found a total prevalence of 6.5%, with the majority of patients older than 75 years being pseudophakic [19], similar to our findings. Given the increasing ageing population and the increase in life expectancy worldwide, the prevalence of cataracts is expected to rise significantly in the coming decades.

### Retina

In the current study, RPE changes were observed in 12% and macular drusen in 3% of the ophthalmic patients, and, as expected, these rates increased with age, from 1.8% to 57% and 0.3% to 24%, in patients 18–40 and over 80 years of age, respectively. Alterations of the RPE are presumed to be important indicators of impaired RPE function [20]. Such RPE changes are an important sign of AMD, such that even minor alterations (e.g., hypopigmentation or RPE elevation) are significant risk factors for progression to exudative AMD [21]. Macular drusen can be a normal sign of aging, especially when they are not associated with RPE changes [22]. The incidence and prevalence of drusen subtypes are known to vary between populations [23]. Early diagnosis of RPE changes and the presence of macular drusen can indicate the need for a prompt ocular computerized tomographic exam for AMD diagnosis, leading to the prevention of vision loss.

### Optic nerve head

The normal ONH varies from one person to another [24]. We observed that the ON had a tilted configuration in 9.2% of our ophthalmic patients, and was more common in females. Other studies described a range of ON tilting to range from 0.09% to 3.5% [25]. Our relatively high rate may be related to the relatively high incidence (30–80%) of myopia in our country’s general population and higher in the ultra-orthodox segment of the population [26]. Moreover, the prevalence and progression of myopia are known to be associated with ethnicity [27].

PPA is highly prevalent in eyes with geographic atrophy due to AMD and also associated with high myopia [28]. The overall prevalence of unspecified PPA in the current study was 13.5%, which is lower than that reported in other studies [29, 30].

Some less common ON disorders were also observed in this study. MNFL is a rare, benign retinal finding that consists of whitish, well-demarcated patches on the outermost retinal surface that obscure the underlying retinal vessels [31]. It has been reported to occur in 0.57% to 1% of the general population, with a potential for growth in 10% of cases [32]. A prevalence of 0.3% was observed in this study.

Optic pit is a rare optic disc cavitary anomaly that is observed in about 1 in 11,000 of the general population [33]. It is typically congenital, unilateral, and occurs equally in males and females [33]. Interestingly, an optic pit was observed in 3 of the 36,762 ophthalmic patients in this study, a very similar prevalence to that described in studies starting as early as 1908 [34]. It can be complicated with optic disc pit maculopathy and require surgical intervention [35].

ON colobomas are rare congenital defects that have been reported to occur in about 0.25% of 12,000 patients examined ophthalmoscopically [36]. Colobomas are due to abnormal closure of the fetal fissure in the inferonasal quadrant of the developing optic cup, and have typical funduscopic appearances [37]. In the current study, ON colobomas were observed in 4 patients, suggesting a higher frequency of 1:9190 patients.

The prevalence of ONHD was 0.1%. It had been reported to be clinically identified in around 1% of patients, subclinical in around 2.4%, and bilateral in most cases [38]. It is typically an incidental asymptomatic finding, however, it can rarely be associated with progressive VF loss, choroidal neovascularization, increased risk of non-arthritic ischemic optic neuropathy, and vascular occlusion [39].

### Limitations

Individuals attending the medical screening institute may not necessarily represent the general population in Israel, with a selection bias towards middle-aged and relatively high-income males who routinely undergo periodic examinations. The study population comprises a self-selected sample rather than a randomized one; participants voluntarily opted for health screening, potentially influenced by their employer or insurer. The decision to attend a health screening may be motivated by existing health concerns, possibly resulting in a higher prevalence of health issues compared to the general population [40]. Conversely, individuals participating in screenings might exhibit heightened health consciousness, prompting earlier detection of health conditions. Furthermore, employment status and possession of health insurance can be associated with better health outcomes [41]. These factors may introduce potential selection biases, such as the healthy worker effect, and confounding by functional status or cognitive impairment [42, 43]. This also explains the high male-to-female ratio, which does not match the near balanced rate found in the general population or in patients attending the ophthalmic emergency room [44].

In conclusion, normal values in medicine serve as reference points for all physical examinations and allow for early detection and monitoring of disease states. Here we present the prevalence of both common and rare ocular findings derived from a very large dataset. With the abovementioned limitations, the values may provide reference points for the diagnosis of diseases and pathological states that comprise the essential elements of a comprehensive ophthalmic assessment. The information presented in this paper may serve as a valuable resource for clinicians, researchers, and policymakers to develop effective strategies for improving eye health and preventing visual impairment.
